# Claudin 18.2 Expression and Outcomes of First-Line Chemoimmunotherapy in HER2-Negative Gastric or Gastroesophageal Junction Cancer: A Single-Center Retrospective Study

**DOI:** 10.3390/curroncol33080460

**Published:** 2026-08-01

**Authors:** Run Bao, Jing Qin, Rong Zhang, Zhuo Xu, Jiahao Liu, Jiaofeng Shen, Shunji Zhang, Yusong Zhang, Hong Zhu, Chunyan Huang, Yan Lu, Tianhua Liu, Wangyang Pu

**Affiliations:** 1 Department of Gastroenterology, Second Affiliated Hospital of Soochow University, Suzhou 215004, China; 13218109835@163.com; 2 Department of Oncology, Second Affiliated Hospital of Soochow University, Suzhou 215004, China; jing955976@163.com (J.Q.); jfshenzz@163.com (J.S.); office_zsj@163.com (S.Z.); w_wenqing1001@163.com (Y.Z.); 3 Department of Respiratory Medicine, Hangzhou First People’s Hospital Tonglu Campus, Hangzhou 311500, China; rongz331@163.com; 4 Department of Oncology, Zhangjiagang First People’s Hospital, Suzhou 215600, China; xuzhuo1070@163.com; 5 Department of Pathology, Second Affiliated Hospital of Soochow University, Suzhou 215004, China; ljh1989316@163.com; 6 Department of Oncology, First Affiliated Hospital of Soochow University, Suzhou 215006, China; zhuhong_jasmine@suda.edu.cn; 7 Department of Chronic Non-Communicable Diseases, Suzhou Center for Disease Control and Prevention, Suzhou 215004, China; 13178847450@163.com (C.H.); mbk@szcdc.cn (Y.L.); 8 Department of Gastrointestinal Surgery, Second Affiliated Hospital of Soochow University, Suzhou 215004, China

**Keywords:** Claudin 18.2, gastric cancer, gastroesophageal junction cancer, PD-L1, chemoimmunotherapy

## Abstract

This study aimed to characterize claudin 18.2 (CLDN18.2) expression in HER2-negative gastric or gastroesophageal junction cancer (GC/GEJC) and to evaluate whether CLDN18.2 status is associated with clinicopathological features and outcomes after first line chemoimmunotherapy. We retrospectively analyzed 189 patients with HER2-negative GC/GEJC treated at our institution from October 2019 to September 2024. CLDN18.2 expression was assessed by immunohistochemistry using two prespecified positivity thresholds: moderate to strong membranous staining (2+) in ≥40% or ≥75% of tumor cells. CLDN18.2 positivity was observed in 92/189 patients (48.7%) using the ≥40% threshold and 69/189 (36.5%) using the ≥75% threshold. PD L1 CPS ≥ 5 was less frequent in CLDN18.2 positive than in CLDN18.2 negative tumors at both thresholds (≥40%: 8.7% vs. 23.7%, *p* = 0.003; ≥75%: 8.7% vs. 20.8%, *p* = 0.019). Among 87 patients receiving first line chemoimmunotherapy, CLDN18.2 status was not associated with significant differences in objective response rate, progression free survival, or overall survival. CLDN18.2 positive tumors showed lower PD L1 expression, but CLDN18.2 status did not identify a subgroup with differential benefit from first line chemoimmunotherapy. These findings suggest that CLDN18.2 status does not appear to serve as a predictive biomarker for immune checkpoint inhibitor-based treatment.

## Simple Summary

Gastric cancer remains difficult to treat, especially when it has spread. A protein called Claudin18.2 is found on many stomach cancer cells and is being studied as a possible target for new drugs. This study looked at 189 patients with HER2-negative gastric cancer to understand whether Claudin18.2 could also help predict how well patients respond to a common treatment: chemotherapy combined with immunotherapy. We found that tumors with Claudin18.2 had lower levels of another protein called PD L1, which is linked to immunotherapy response. However, Claudin18.2 status did not affect treatment outcomes—patients with or without this protein had similar survival and response rates. These findings suggest that Claudin18.2 is more useful as a direct drug target rather than as a test to guide immunotherapy choices.

## 1. Introduction

Gastric or gastroesophageal junction cancer (GC/GEJC) is the fifth most common cancer and the fourth leading cause of cancer-related death worldwide [1]. Fluoropyrimidine plus platinum chemotherapy remains the backbone of systemic treatment for metastatic GC/GEJC. HER2-targeted therapy and anti-PD-1 antibodies combined with chemotherapy have improved first-line outcomes in selected molecular subgroups, particularly HER2-positive, PD-L1-enriched, or microsatellite instability-high tumors [2,3]. However, many patients have limited or short-lived benefit, and additional therapeutic targets are needed.

Claudin 18.2 (CLDN18.2), a tight-junction protein normally restricted to gastric mucosal epithelial cells, can become exposed on the tumor-cell surface during malignant transformation, making it an attractive and relatively specific therapeutic target in GC/GEJC [4]. Upon malignant transformation, loss of cellular polarity exposes CLDN18.2 epitopes, rendering the protein accessible to antibody-based therapeutics [5,6]. The phase II FAST trial first showed that adding zolbetuximab to EOX chemotherapy improved PFS and OS in advanced CLDN18.2-positive G/GEJ adenocarcinoma defined as ≥2+ staining in ≥40% of tumor cells [7]. The phase III SPOTLIGHT and GLOW trials subsequently confirmed survival benefits with zolbetuximab plus chemotherapy in HER2-negative, CLDN18.2-positive disease using the more stringent threshold of ≥2+ staining in ≥75% of tumor cells [8,9]. The markedly different CLDN18.2 positivity thresholds used across the pivotal trials illustrate a conceptual shift toward more stringent patient selection, although head-to-head comparisons of the clinical and biological profiles defined by each threshold are lacking. Beyond monoclonal antibodies, CLDN18.2-directed antibody-drug conjugates and CAR T-cell therapy have shown early clinical activity [10,11,12]. With CLDN18.2-targeted treatment entering clinical practice, the prevalence, clinicopathological correlates, and interaction of CLDN18.2 with established biomarkers such as PD-L1, EBV, and MMR status require further clarification.

Most prior work has focused on CLDN18.2 as a therapeutic target, whereas its predictive role in immunotherapy is less defined. CLDN18.2 expression has been linked to PD-L1 and may influence the immune microenvironment, raising the possibility of an association with immunotherapy response [13,14]. However, the biological basis of this link remains poorly understood: it is unclear whether the lower PD-L1 levels observed in CLDN18.2-positive tumors reflect an intrinsic molecular feature of the tumor microenvironment or point to an alternative immune evasion mechanism independent of the PD-L1/PD-1 axis [15]. In particular, whether CLDN18.2 expression predicts response to immune checkpoint inhibitor-based first-line therapy remains uncertain.

To address these questions, we retrospectively analyzed a Chinese cohort of HER2-negative GC/GEJC patients, applying the two clinically relevant CLDN18.2 positivity thresholds (≥2+ in ≥40% and ≥75% of tumor cells). Associations between CLDN18.2 expression and clinicopathological or molecular features were evaluated, along with treatment outcomes in patients receiving first-line chemoimmunotherapy.

## 2. Materials and Methods

### 2.1. Study Design and Patients

This single-center retrospective study enrolled patients with HER2-negative GC/GEJC who underwent surgery or received systemic therapy at the Second Affiliated Hospital of Soochow University between October 2019 and September 2024. Eligible patients were 18–75 years of age, had histologically confirmed adenocarcinoma, and had available pretreatment primary tumor specimens with CLDN18.2 IHC results. Patients with HER2-positive disease were excluded. In addition, patients with insufficient clinical data for outcome evaluation were not included. Clinical data, including demographic characteristics, tumor stage, histopathological features, treatment regimens, and survival outcomes, were extracted from electronic medical records using a standardized case report form. Written informed consent was obtained for biomarker testing. The study protocol was approved by the Ethics Committee of the Second Affiliated Hospital of Soochow University (approval number: LK2024059).

### 2.2. Molecular Characterization

All molecular analyses were performed on formalin-fixed, paraffin-embedded (FFPE) tumor tissue sections. Immunohistochemistry (IHC) was performed using an automated staining platform according to standard protocols. CLDN18.2 expression was evaluated by IHC with the 43-14A antibody (Roche Ventana, Tucson, AZ, USA). Positivity was defined using two prespecified thresholds: moderate-to-strong (2+) membranous staining in ≥40% or ≥75% of tumor cells. PD-L1 expression was assessed with the 22C3 pharmDx assay (DAKO, Carpinteria, CA, USA) and reported as the CPS, calculated as (number of PD-L1-positive tumor cells, lymphocytes, and macrophages/total number of viable tumor cells) × 100; CPS ≥ 5 was considered positive. HER2 status was determined by IHC with the 4B5 antibody (Roche, Tucson, AZ, USA), and cases scored 3+ or 2+ with confirmatory in situ hybridization were considered HER2-positive. EBV status was detected by EBER in situ hybridization (Ventana INFORM EBER Probe, Ventana Medical Systems, Tucson, AZ, USA). MMR status was evaluated by IHC for MLH1, MSH2, MSH6, and PMS2 (GeneTech, Shanghai, China); loss of nuclear expression of any protein was classified as deficient MMR (dMMR), whereas retained expression of all four proteins was classified as proficient MMR (pMMR). All pathological assessments were independently reviewed by two experienced pathologists who were blinded to clinical outcomes. Discrepancies were resolved by consensus discussion. The scoring of CLDN18.2 positivity was based on membranous staining intensity and the percentage of positive tumor cells, assessed in at least five representative high-power fields at ×200 magnification. Representative IHC images illustrating CLDN18.2 staining at both positivity thresholds are provided in Figure S1 (Supplementary Materials).

### 2.3. Outcomes and Statistical Analysis

Objective response rate (ORR), progression-free survival (PFS), and overall survival (OS) were the primary efficacy endpoints. Tumor responses were evaluated per RECIST version 1.1 in patients with measurable disease at baseline. PFS was measured from the start of first-line chemoimmunotherapy to disease progression or death. OS was measured from treatment initiation to death, with censoring at the last follow-up for patients without an event. All analyses were performed on available data only. Categorical variables were compared using the chi-square test or Fisher’s exact test, as appropriate. Survival curves were estimated using the Kaplan–Meier method and compared with the log-rank test. The proportional hazards assumption for Cox regression models was tested using Schoenfeld residuals and was satisfied for all covariates included in the final models. Statistical analyses were conducted using SPSS (version 21.0; IBM Corp.) and jamovi (version 2.6.22). Two-sided *p* values < 0.05 were considered statistically significant. Univariate and multivariate Cox proportional hazards models were used to estimate hazard ratios (HRs) and 95% confidence intervals (CIs), with multivariate models including eight pre-specified covariates based on clinical relevance and known prognostic factors in gastric cancer. The proportional hazards assumption was tested using Schoenfeld residuals and was satisfied for all covariates in the final models. Given the exploratory nature of these analyses, we did not adjust for multiple comparisons.

## 3. Results

A total of 189 patients were included (median age, 69 years; 139/189 [73.5%] male). Tumor specimens consisted of 77 endoscopic biopsies and 112 surgical resections. Using the ≥40% threshold, CLDN18.2 positivity was observed in 92 patients (48.7%), broadly consistent with the FAST trial. Using the ≥75% threshold, 69 patients (36.5%) were CLDN18.2-positive.

At both thresholds, CLDN18.2-positive tumors had a significantly lower prevalence of PD-L1 CPS ≥ 5 than CLDN18.2-negative tumors (≥40% threshold: 8.7% vs. 23.7%, *p* = 0.003; ≥75% threshold: 8.7% vs. 20.8%, *p* = 0.019). No significant differences were observed by age, sex, specimen type, histological type, differentiation, stage, MMR status, EBV status, or HER2 expression category (Table 1 and Table 2).

**Figure 1:**
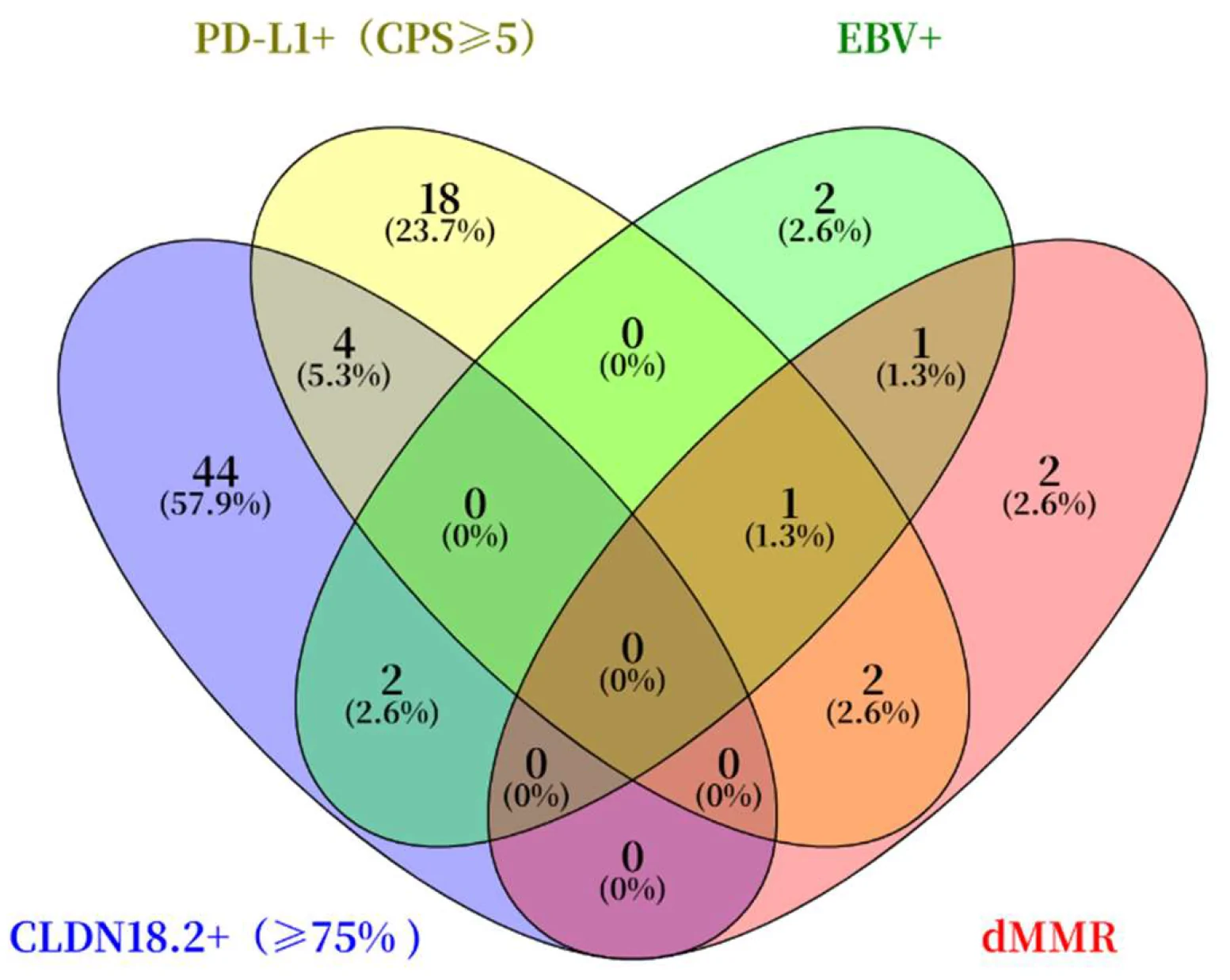
Co-expression landscape of CLDN18.2 with PD-L1, EBV, and MMR status in HER2-negative GC/GEJC. *Venn diagram showing overlap among CLDN18.2 positivity (≥75% threshold), PD-L1 CPS ≥ 5, EBV positivity, and dMMR status. Among 135 evaluable patients, 50 (37.0%) were CLDN18.2-positive, 25 (18.5%) had PD-L1 CPS ≥ 5, 6 (4.4%) were EBV-positive, and 6 (4.4%) had dMMR. Overlaps included CLDN18.2/PD-L1 (n = 4) and CLDN18.2/EBV (n = 2). Fifty-nine patients had none of these positive biomarkers*. Note: percentages displayed within the diagram are calculated relative to the biomarker-positive subset (*n* = 76), while the prevalence estimates in this caption refer to the total evaluable cohort (*n* = 135).

Among the 189 patients, 135 had evaluable results for all four biomarkers included in the co-expression analysis: CLDN18.2 (using the ≥75% threshold), PD-L1 CPS, EBV status, and MMR status. The distribution and overlap of these biomarkers are shown in Figure 1.

Among the 189 patients, 87 received first-line chemoimmunotherapy. None received zolbetuximab, reflecting real-world practice during the study period. Baseline characteristics by CLDN18.2 status using the ≥40% and ≥75% thresholds are shown in Table 3 and Table 4. Median follow-up in this subgroup was 15.3 months. Three patients lacked evaluable lesions and were excluded from ORR and PFS analyses; therefore, 84 patients were response-evaluable. ORR did not differ significantly between CLDN18.2-negative and -positive groups at either threshold (≥40%: 20/45 [44.4%] vs. 13/39 [33.3%], *p* = 0.298; ≥75%: 26/61 [42.6%] vs. 7/23 [30.4%], *p* = 0.308).

**Figure 2:**
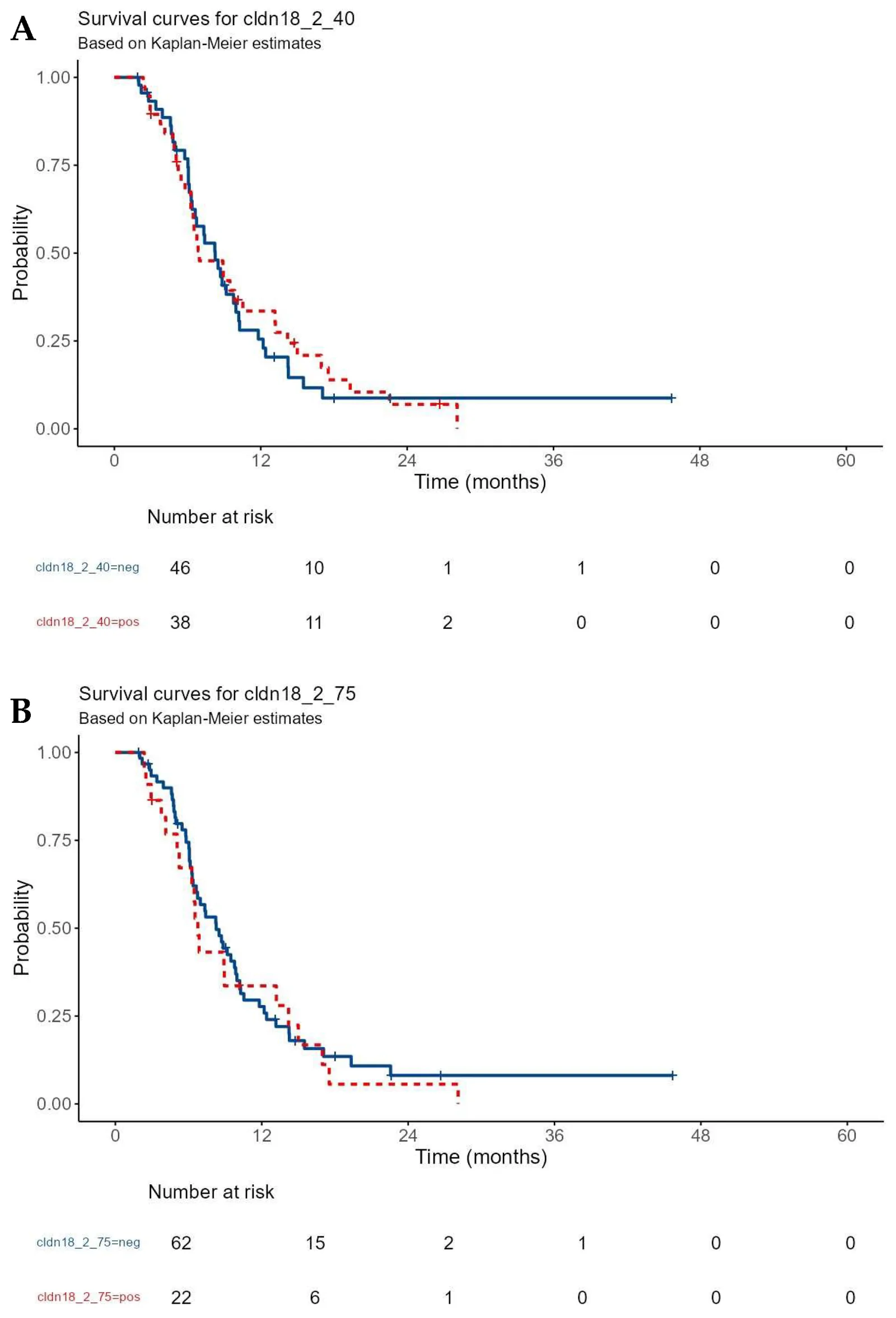
Progression-free survival according to CLDN18.2 expression status. *Kaplan–Meier curves showing PFS in 84 patients receiving first-line chemoimmunotherapy, stratified by CLDN18.2 status using the ≥40% threshold (**A**) and the ≥75% threshold (**B**). p values were calculated with the log-rank test.*

**Figure 3:**
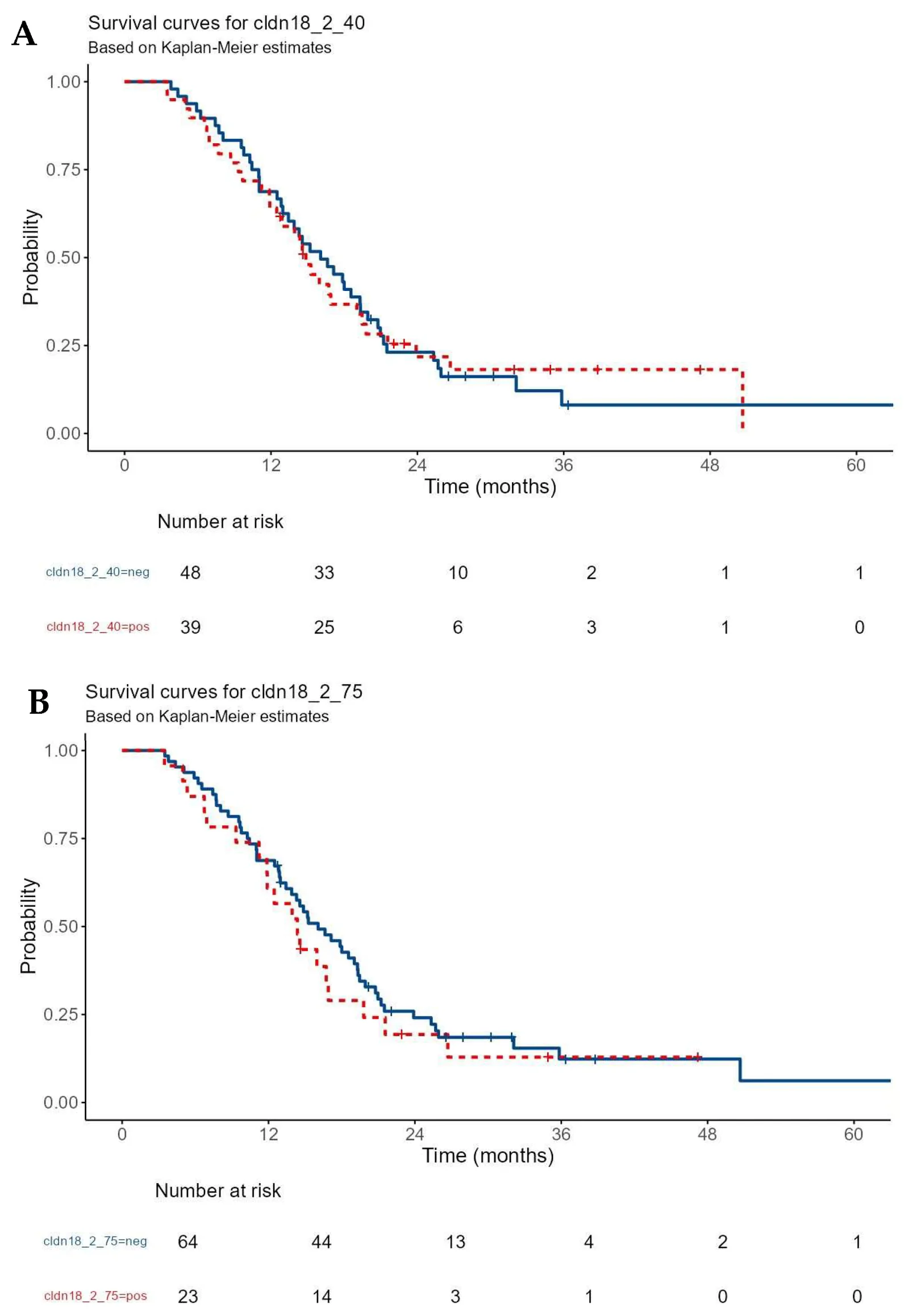
Overall survival according to CLDN18.2 expression status. *Kaplan–Meier curves showing OS in 87 patients receiving first-line chemoimmunotherapy, stratified by CLDN18.2 status using the ≥40% threshold (**A**) and the ≥75% threshold (**B**). p values were calculated with the log-rank test.*

Survival outcomes were also comparable by CLDN18.2 status. Using the ≥40% threshold, median PFS was 8.27 months (95% CI, 6.37–10.2) in CLDN18.2-negative patients and 6.97 months (95% CI, 6.27–13.2) in CLDN18.2-positive patients (HR, 0.95; 95% CI, 0.59–1.52; *p* = 0.828). Median OS was 16.1 months (95% CI, 13.4–19.9) and 14.9 months (95% CI, 12.5–19.8), respectively (HR, 1.00; 95% CI, 0.63–1.60; *p* = 0.997) (Figure 2A and Figure 3A). Using the ≥75% threshold, median PFS was 8.27 months (95% CI, 6.63–10.2) in CLDN18.2-negative patients and 6.77 months (95% CI, 5.23–15.0) in CLDN18.2-positive patients (HR, 1.15; 95% CI, 0.68–1.94; *p* = 0.599). Median OS was 16.1 months (95% CI, 13.4–19.5) and 14.4 months (95% CI, 11.9–21.6), respectively (HR, 1.19; 95% CI, 0.71–2.02; *p* = 0.507) (Figure 2B and Figure 3B).

Multivariate Cox models adjusting for sex, age, tumor location, HER2 expression category, PD-L1 CPS, EBV status, and MMR status showed that CLDN18.2 status was not independently associated with PFS or OS at either positivity threshold (Table 5 and Table 6).

## 4. Discussion

The clinical significance of CLDN18.2 in GC/GEJC remains incompletely defined because published studies differ in disease stage, sampling method, antibody clone, staining platform, and positivity threshold [16,17,18,19,20,21]. This heterogeneity has contributed to variation in CLDN18.2 positivity rates and inconsistent findings regarding its association with PD-L1, EBV, and MMR across studies [22,23]. In this single-center Chinese cohort of 189 patients with HER2-negative GC/GEJC, we applied both the FAST-derived threshold (≥2+ staining in ≥40% of tumor cells) and the SPOTLIGHT/GLOW threshold (≥2+ staining in ≥75% of tumor cells) to systematically evaluate how threshold selection affects CLDN18.2 expression distribution and its associations with clinicopathological and molecular features. The main findings were that CLDN18.2 positivity was common, was associated with lower PD-L1 CPS ≥ 5 prevalence, and was not associated with ORR, PFS, or OS among patients receiving first-line chemoimmunotherapy.

The CLDN18.2 positivity rate in our cohort was 48.7% using the ≥40% threshold, within the range reported in the FAST trial and Asian real-world series [7,17,19,24]. When the ≥75% threshold was applied, the rate decreased to 36.5%, close to the 38.4% prevalence reported across SPOTLIGHT and GLOW and within the range of other contemporary studies using similar criteria [16,17,18,20,25]. Differences across studies are likely driven by patient ethnicity, disease stage, sampling site, antibody clone, staining platform, and scoring threshold [25,26,27,28]. These factors underscore the need for standardized CLDN18.2 testing and careful reporting of the exact assay and cutoff used.

We did not observe statistically significant associations between CLDN18.2 status and sex, age, Lauren classification, tumor location, differentiation, or stage. Although several studies have linked CLDN18.2 expression with diffuse-type histology, others using contemporary diagnostic antibodies and thresholds have reported broadly similar positivity across Lauren subtypes [16,18,25,29]. Our data therefore support testing for CLDN18.2 regardless of histological subtype, particularly as CLDN18.2-targeted therapies are considered for HER2-negative disease.

Biopsy and surgical specimens showed comparable CLDN18.2 positivity rates in our cohort. This is clinically relevant because treatment decisions in advanced GC/GEJC often rely on endoscopic biopsy material. Prior analyses using virtual biopsies and paired specimens suggest that CLDN18.2 assessment is affected by intratumoral heterogeneity, but diagnostic sensitivity improves as the number of biopsy fragments increases, with limited incremental gain beyond approximately six fragments [16,25]. When CLDN18.2 testing is performed on biopsy material, adequate sampling and careful pathological review remain essential.

An important finding of our study was the lower prevalence of PD-L1 CPS ≥ 5 among CLDN18.2-positive tumors at both thresholds. In SPOTLIGHT and GLOW, PD-L1 CPS ≥ 5 was reported in 13.2% and 21.9% of CLDN18.2-positive tumors, respectively, lower than the proportions reported in some first-line immunotherapy trials enrolling patients irrespective of CLDN18.2 status [2,3,8,9,25]. However, retrospective cohorts using similar CLDN18.2 assays have not consistently confirmed an inverse association between CLDN18.2 and PD-L1 expression [16,18]. The lower PD-L1 positivity in our cohort may reflect biological differences, such as an immunologically less inflamed tumor microenvironment, or cohort-specific factors, including referral patterns, local PD-L1 prescreening, and missing PD-L1 data. The inverse CLDN18.2–PD-L1 association may reflect alternative immune evasion mechanisms: CLDN18.2-positive tumors appear to rely on TGF-β-driven stromal remodeling and regulatory T-cell infiltration rather than the PD-L1/PD-1 axis [14,15]. Single cell data have linked these tumors to galectin 3 CD44 signaling and expanded Treg populations, implying less dependence on PD L1 mediated suppression [14]. This is consistent with a 563 case study showing negative correlation between CLDN18.2 and PD L1 expression, supporting the immune cold phenotype of CLDN18.2 positive gastric cancer [30]. These possibilities should be tested in larger cohorts with paired immune-microenvironment profiling.

We did not identify a significant association between CLDN18.2 status and EBV or MMR status. Earlier studies suggested enrichment of CLDN18.2 expression in EBV-positive tumors, whereas more recent advanced-disease cohorts have shown similar CLDN18.2 prevalence across EBV and MMR subgroups [17,18,20,25,29,31]. Our findings align with recent evidence and further support that CLDN18.2 expression is not strongly associated with EBV or MMR status in advanced GC/GEJC. In our cohort, the small numbers of EBV-positive and dMMR tumors limited statistical power; therefore, absence of association should be interpreted cautiously.

Despite lower PD-L1 expression in CLDN18.2-positive tumors, first-line chemoimmunotherapy produced comparable ORR, PFS, and OS in CLDN18.2-positive and -negative patients. This finding suggests that CLDN18.2 status alone should not be used to exclude patients from immune checkpoint inhibitor-based first-line therapy. Our results are consistent with the study by Kim et al., in which outcomes with first-line nivolumab plus chemotherapy did not differ by CLDN18.2 status using the ≥75% threshold [20], and with the broader clinical and molecular analysis by Kubota et al., which found no clear impact of CLDN18.2 on chemotherapy or anti-PD-1 outcomes [18]. In contrast, Qi et al. reported poorer immunotherapy-related PFS and OS in CLDN18.2-positive advanced gastric cancer [17]. Differences in treatment line, PD-1 inhibitor exposure, threshold selection (≥70% vs. ≥75%), sample size, and immune contexture may explain these divergent results. Our cohort adds China-specific real-world data in the first-line chemoimmunotherapy setting and includes both clinically relevant CLDN18.2 cutoffs.

In multivariate analyses, CLDN18.2 was not independently associated with OS or PFS. Age ≥ 65 years was associated with worse survival, whereas PD-L1 CPS ≥ 5 was associated with a lower risk of death in the model using the ≥75% CLDN18.2 threshold, with a similar trend in the ≥40% model. These findings are directionally consistent with first-line immunotherapy trials and meta-analyses showing greater benefit from PD-1 blockade in PD-L1-enriched gastric and gastroesophageal junction cancers [2,3,32,33]. The consistency of this PD-L1 effect across both threshold models adds internal validity to our findings. Because the treated subgroup was small, these covariate associations should be viewed as exploratory rather than definitive.

The optimal first-line strategy for HER2-negative, CLDN18.2-positive GC/GEJC remains undefined. Zolbetuximab plus chemotherapy has demonstrated consistent survival benefits in the FAST, SPOTLIGHT, and GLOW trials [7,8,9], yet direct head-to-head comparisons with PD-1 inhibitor-based chemoimmunotherapy are lacking, and real-world evidence for zolbetuximab-containing regimens remains limited. For patients with CLDN18.2-positive and PD-L1-low tumors, a CLDN18.2-targeted approach may be a reasonable choice, given the modest benefit of immune checkpoint inhibitors in this subgroup. Nevertheless, treatment decisions must integrate multiple factors, including dMMR/MSI-H status, EBV positivity, performance status, toxicity profile, reimbursement, and planned treatment sequence [18,27,28,29,30]. Future research should focus on developing integrated biomarker algorithms that combine CLDN18.2 expression, PD-L1 CPS, MMR/EBV status, genomic alterations, and digital-pathology or AI-derived immune features to optimize patient selection and treatment sequencing [31].

### Limitations

This study has some limitations. First, the single-center retrospective design is subject to selection and information biases that cannot be fully adjusted, which may limit the generalizability of our conclusions. Second, our eligibility criteria excluded patients over 75 years of age, which may limit the generalizability of our findings to older patients, who constitute a substantial proportion of the gastric cancer population. Third, the modest sample size of the chemoimmunotherapy subgroup (*n* = 87) may have limited the statistical power of our multivariable analyses, particularly for subgroup assessments involving rare events or low-prevalence biomarkers such as dMMR or EBV positivity. Given the limited event count, the eight-covariate Cox model may be overfitted, as suggested by the wide confidence intervals for certain estimates. These findings should therefore be considered exploratory and hypothesis-generating, warranting validation in larger independent cohorts.

An additional methodological concern relates to the use of archival primary tumor specimens for CLDN18.2 IHC. Preanalytical variables, including fixation duration, tissue processing, and block age, may affect IHC sensitivity and introduce variability in biomarker classification. Although all samples were processed according to standardized institutional protocols, the impact of these factors cannot be entirely excluded. Additionally, we did not evaluate biopsy-resection concordance for CLDN18.2; given its intratumoral heterogeneity, biopsy-based classification may not fully capture the overall tumor status, despite prior reports that approximately six fragments may suffice. Regarding biomarker assessment, PD-L1 expression data were incomplete for a small subset of patients, and our analysis was restricted to a single CPS cutoff (≥5). While clinically relevant, this threshold did not permit evaluation of potential dose–response relationships between PD-L1 levels and treatment outcomes. In addition, the heterogeneity in PD-1 inhibitor selection and chemotherapy backbones—while reflective of real-world practice—limited regimen-specific subgroup analyses; however, this also enhances the generalizability of our findings. Finally, the absence of a chemotherapy-alone control arm precludes a definitive assessment of whether CLDN18.2 expression predicts incremental benefit from immune checkpoint inhibitors.

## 5. Conclusions

In this HER2-negative GC/GEJC cohort, CLDN18.2 expression was common and inversely associated with PD-L1 CPS ≥ 5. However, CLDN18.2 status was not associated with ORR, PFS, or OS in patients receiving first-line chemoimmunotherapy, nor was it an independent prognostic factor after adjustment for clinicopathological variables and other biomarkers. These findings suggest that CLDN18.2 status does not appear to serve as a predictive biomarker for clinical outcomes in the chemoimmunotherapy treated population. Prospective studies with standardized CLDN18.2 assays and integrated biomarker-driven stratification are needed to guide first-line treatment selection in this population.

## Figures and Tables

**Table 1:** Clinicopathological and molecular characteristics stratified by CLDN18.2 status (cutoff: ≥2+ in ≥40% of tumor cells).

	*n* (%)		
Characteristics	CLDN18.2+ (*n* = 92, 48.7%)	CLDN18.2− (*n* = 97, 51.3%)	*p* Value
Age, years			
<65	34 (37.0%)	31 (32.0%)	
≥65	58 (63.0%)	66 (68.0%)	0.47
Sex			
Male	63 (68.5%)	76 (78.4%)	
Female	29 (31.5%)	21 (21.6%)	0.124
Histological type			
Intestinal	11 (11.9%)	5 (5.2%)	
Diffuse	17 (18.5%)	12 (12.4%)	
Mixed	25 (27.2%)	26 (26.8%)	0.348
Not available	39 (42.4%)	54 (55.6%)	
Primary tumor location at initial diagnosis			
Gastroesophageal junction cancer	20 (21.7%)	29 (29.9%)	0.201
Gastric cancer	72 (78.3%)	68 (70.1%)	
Differentiation			
Moderate	7 (7.6%)	5 (5.2%)	0.829
Moderate-poor	15 (16.3%)	13 (13.4%)	
Poor	59 (64.1%)	59 (60.8%)	
Not available	11 (12.0%)	20 (20.6%)	
MMR			
pMMR	89 (96.7%)	92 (94.8%)	0.518
dMMR	3 (3.3%)	5 (5.2%)	
EBV			
Positive	4 (4.3%)	2 (2.1%)	0.312
Negative	63 (68.5%)	75 (77.3%)	
Not available	25 (27.2%)	20 (20.6%)	
HER2			
2+/FISH-	7 (7.6%)	7 (7.2%)	0.897
≤1+	80 (87.0%)	86 (88.7%)	
Not available	5 (5.4%)	4 (4.1%)	
PD-L1			
<5	81 (88.0%)	67 (69.1%)	0.003
≥5	8 (8.7%)	23 (23.7%)	
Not available	3 (3.3%)	7 (7.2%)	
Stage			
I-II	4 (4.4%)	7 (7.2%)	0.400
III-IV	88 (95.6%)	90 (92.8%)	
Specimen type			
Biopsy	33 (35.9%)	44 (45.4%)	0.184
Surgical resection	59 (64.1%)	53 (54.6%)	

**Table 2:** Clinicopathological and molecular characteristics stratified by CLDN18.2 status (cutoff: ≥2+ in ≥75% of tumor cells).

	*n* (%)		
Characteristics	CLDN18.2+ (*n* = 69, 36.5%)	CLDN18.2− (*n* = 120, 63.5%)	*p* Value
Age, years			
<65	26 (37.7%)	40 (33.3%)	0.565
≥65	43 (62.3%)	80 (66.7%)	
Sex			
Male	45 (65.2%)	94 (78.3%)	0.060
Female	24 (34.8%)	26 (21.7%)	
Histological type			
Intestinal	8 (11.6%)	8 (6.7%)	0.721
Diffuse	13 (18.8%)	16 (13.3%)	
Mixed	20 (29.0%)	31 (25.8%)	
Not available	28 (40.6%)	65 (54.2%)	
Primary tumor location at initial diagnosis			
Gastroesophageal junction cancer	17 (24.6%)	32 (26.7%)	0.759
Gastric cancer	52 (75.4%)	88 (73.3%)	
Differentiation			
Moderate	7 (10.1%)	5 (4.2%)	0.302
Moderate-poor	9 (13.0%)	19 (15.8%)	
Poor	48 (69.6%)	70 (58.3%)	
Not available	5 (7.2%)	26 (21.7%)	
MMR			
pMMR	68 (98.6%)	113 (94.2%)	0.15
dMMR	1 (1.4%)	7 (5.8%)	
EBV			
Positive	2 (2.9%)	4 (3.3%)	0.942
Negative	48 (69.6%)	90 (75.0%)	
Not available	19 (27.5%)	26 (21.7%)	
HER2			
2+/FISH-	3 (4.3%)	11 (9.2%)	0.234
≤1+	62 (89.9%)	104 (86.7%)	
Not available	4 (5.8%)	5 (4.2%)	
PD-L1			
<5	62 (89.9%)	86 (71.7%)	0.019
≥5	6 (8.7%)	25 (20.8%)	
Not available	1 (1.4%)	9 (7.5%)	
Stage			
I-II	4 (5.8%)	7 (5.8%)	1.000
III-IV	65 (94.2%)	113 (94.2%)	
Specimen type			
Biopsy	23 (23.3%)	54 (45.0%)	0.116
Surgical resection	46 (66.7%)	66 (55.0%)	

**Table 3:** Baseline characteristics of patients receiving first-line chemoimmunotherapy stratified by CLDN18.2 status (cutoff: ≥2+ in ≥40% of tumor cells).

	*n* (%)		
Characteristics	CLDN18.2− (*n* = 48)	CLDN18.2+ (*n* = 39)	*p* Value
Age, years			
<65	10 (20.8%)	17 (43.6%)	
≥65	38 (79.2%)	22 (56.4%)	0.023
Sex			
Male	35 (72.9%)	27 (69.2%)	
Female	13 (27.1%)	12 (30.8%)	0.706
Histological type			
Mixed	6 (12.5%)	5 (12.8%)	
Intestinal	1 (2%)	4 (10.3%)	
Diffuse	3 (6.3%)	4 (10.3%)	0.335
Not available	38 (79.2%)	26 (66.7%)	
Primary tumor location at initial diagnosis			
Gastroesophageal junction cancer	18 (37.5%)	6 (15.4%)	0.022
Gastric cancer	30 (62.5%)	33 (84.6%)	
Differentiation			
Moderate	28 (58.3%)	24 (61.5%)	0.412
Moderate-poor	3 (6.3%)	6 (15.4%)	
Poor	2 (4.2%)	1 (2.6%)	
Not available	15 (31.3%)	8 (20.5%)	
MMR			
pMMR	46 (95.8%)	38 (97.4%)	0.684
dMMR	2 (4.2%)	1 (2.6%)	
EBV			
Positive	2 (4.2%)	1 (2.6%)	0.909
Negative	31 (64.6%)	25 (64.1%)	
Not available	15 (31.3%)	13 (33.3%)	
PD-L1			
<5	31 (64.6%)	34 (87.2%)	0.024
≥5	13 (27.1%)	2 (5.1%)	
Not available	4 (8.3%)	3 (7.7%)	
Prior gastrectomy			
Yes	8 (16.7%)	12 (30.8%)	0.12
No	40 (83.3%)	27 (69.2%)	
Immunotherapy			
Nivolumab	6 (12.5%)	6 (15.4%)	0.925
Tislelizumab	6 (12.5%)	4 (10.3%)	
Sintilimab	28 (58.3%)	24 (61.5%)	
Others	8 (16.7%)	5 (12.8%)	
Chemotherapy			
Oxaliplatin plus capecitabine/S-1	25 (52.1%)	21 (53.8%)	0.716
Paclitaxel plus capecitabine/S-1	20 (41.7%)	17 (43.6%)	
S-1	3 (6.3%)	1 (2.6%)	

**Table 4:** Baseline characteristics of patients receiving first-line chemoimmunotherapy stratified by CLDN18.2 status (cutoff: ≥2+ in ≥75% of tumor cells).

	*n* (%)		
Characteristics	CLDN18.2− (*n* = 64)	CLDN18.2+ (*n* = 23)	*p* Value
Histological type			
Mixed	9 (14.1%)	2 (8.7%)	
Intestinal	3 (4.7%)	2 (8.7%)	
Diffuse	5 (7.8%)	2 (8.7%)	0.831
Not available	47 (73.4%)	17 (73.9%)	
Primary tumor location at initial diagnosis			
Gastroesophageal junction cancer	21 (32.8%)	3 (13.0%)	0.069
Gastric cancer	43 (67.2%)	20 (87.0%)	
Differentiation			
Moderate	34 (53.1%)	18 (78.3%)	0.129
Moderate-poor	7 (10.9%)	2 (8.7%)	
Poor	2 (3.1%)	1 (4.3%)	
Not available	21 (32.8%)	2 (8.7%)	
MMR			
pMMR	61 (95.3%)	23 (100%)	0.291
dMMR	3 (4.7%)	0	
EBV status			
Positive	3 (4.7%)	0	0.563
Negative	41 (64.1%)	15 (65.2%)	
Not available	20 (31.3%)	8 (34.8%)	
PD-L1 status			
<5	45 (70.3%)	20 (87.0%)	0.289
≥5	13 (20.3%)	2 (8.7%)	
Not available	6 (9.4%)	1 (4.3%)	
Prior gastrectomy			
Yes	15 (23.4%)	5 (21.7%)	0.868
No	49 (76.6%)	18 (78.3%)	
Immunotherapy			
Nivolumab	9 (14.1%)	3 (13.0%)	0.619
Tislelizumab	6 (9.4%)	4 (17.4%)	
Sintilimab	38 (59.4%)	14 (60.9%)	
Others	11 (17.2%)	2 (8.7%)	
Chemotherapy			
Oxaliplatin plus capecitabine/S-1	33 (51.6%)	13 (56.5%)	0.199
Paclitaxel plus capecitabine/S-1	28 (43.8%)	9 (39.1%)	
S-1	3 (4.7%)	1 (4.3%)	

**Table 5:** Multivariate Cox regression analysis for progression-free survival.

Parameters	HR (95% CI)	*p* Value
CLDN18.2 cutoff ≥ 2+, 40%		
Male vs. Female	0.72 (0.33–1.59)	0.416
Age ≥ 65 vs. <65 years	2.44 (1.13–5.29)	0.023
GEJ vs. gastric cancer	0.76 (0.37–1.56)	0.454
CLDN18.2-negative vs. positive	0.67 (0.36–1.25)	0.209
HER2 1+/0 vs. HER2 2+/FISH-	0.48 (0.18–1.30)	0.150
PD-L1 CPS ≥ 5 vs. <5	0.41 (0.16–1.02)	0.054
EBV-negative vs. positive	0.45 (0.09–2.27)	0.331
dMMR vs. pMMR	0.81 (0.16–4.03)	0.798
CLDN18.2 cutoff ≥ 2+, 75%		
Male vs. Female	0.74 (0.33–1.67)	0.473
Age ≥ 65 vs. <65 years	2.57 (1.17–5.64)	0.018
GEJ vs. gastric cancer	0.74 (0.36–1.52)	0.411
CLDN18.2-negative vs. positive	1.29 (0.63–2.64)	0.481
HER2 1+/0 vs. HER2 2+/FISH-	0.40 (0.14–1.10)	0.075
PD-L1 CPS ≥ 5 vs. <5	0.39 (0.15–1.02)	0.055
EBV-negative vs. positive	0.62 (0.13–3.02)	0.557
dMMR vs. pMMR	0.71 (0.13–3.79)	0.691

**Table 6:** Multivariate Cox regression analysis for overall survival.

Parameters	HR (95% CI)	*p* Value
CLDN18.2 cutoff ≥ 2+, 40%		
Male vs. Female	1.13 (0.51–2.47)	0.766
Age ≥ 65 vs. <65 years	2.39 (1.08–5.28)	0.031
GEJ vs. gastric cancer	1.09 (0.52–2.26)	0.826
CLDN18.2-negative vs. positive	0.90 (0.46–1.74)	0.745
HER2 1+/0 vs. HER2 2+/FISH-	0.38 (0.14–1.07)	0.066
PD-L1 CPS ≥ 5 vs. <5	0.43 (0.18–1.03)	0.058
EBV-negative vs. positive	0.49 (0.09–2.63)	0.402
dMMR vs. pMMR	0.89 (0.18–4.42)	0.887
CLDN18.2 cutoff ≥ 2+, 75%		
Male vs. Female	1.15 (0.52–2.53)	0.728
Age ≥ 65 vs. <65 years	2.76 (1.20–6.36)	0.017
GEJ vs. gastric cancer	1.05 (0.50–2.20)	0.89
CLDN18.2-negative vs. positive	1.37 (0.62–3.03)	0.441
HER2 1+/0 vs. HER2 2+/FISH-	0.63 (0.25–1.59)	0.326
PD-L1 CPS ≥ 5 vs. <5	0.40 (0.16–0.98)	0.046
EBV-negative vs. positive	0.54 (0.10–2.91)	0.474
dMMR vs. pMMR	0.85 (0.17–4.37)	0.848

## Data Availability

The data presented in this study are available on request from the corresponding author due to patient privacy and ethical restrictions.

## Supplementary Materials

The following supporting information can be downloaded at: https://www.mdpi.com/article/10.3390/curroncol33080460/s1, Figure S1: Representative immunohistochemical staining of CLDN18.2 in HER2‑negative gastric cancer..

## Abbreviations

The following abbreviations are used in this manuscript:

ADCs	Antibody-drug conjugates
CAR-T	Chimeric antigen receptor T cells
CIs	Confidence intervals
CLDN18.2	Claudin 18.2
CPS	Combined positive score
dMMR	Deficient mismatch repair
EBV	Epstein–Barr virus
FFPE	Formalin-fixed, paraffin-embedded
GC/GEJC	Gastric or gastroesophageal junction cancers
HRs	Hazard ratios
IHC	Immunohistochemistry
MMR	Mismatch repair
ORR	Objective response rate
OS	Overall survival
pMMR	Proficient mismatch repair
PFS	Progression-free survival
