# Supplementary material for: Claudin 18.2 Expression and Outcomes of First-Line Chemoimmunotherapy in HER2-Negative Gastric or Gastroesophageal Junction Cancer: A Single-Center Retrospective Study
Source: Curr Oncol. 2026 Aug 1;33(8):460. doi: 10.3390/curroncol33080460 (PMC13510881; doi:10.3390/curroncol33080460)
Supplement: Supplementary file 1 [file curroncol-33-00460-s001.zip › curroncol-4398391-supplementary.pdf]

Figure S1. Representative immunohistochemical staining of CLDN18.2 in HER2-negative gastric cancer.

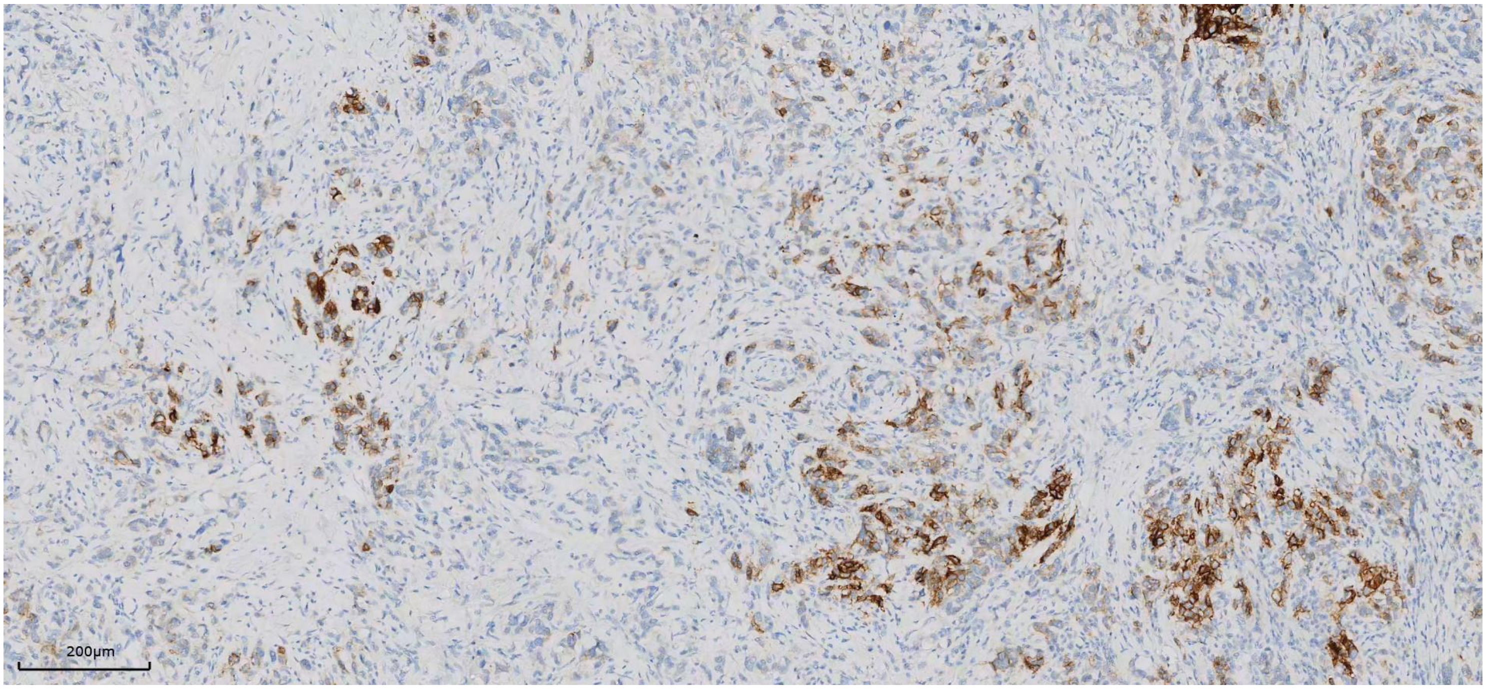

(A) Case meeting the ⩾40% threshold: ⩾2+ membranous CLDN18.2 staining in ⩾40% of tumor cells (original magnification, ×200).

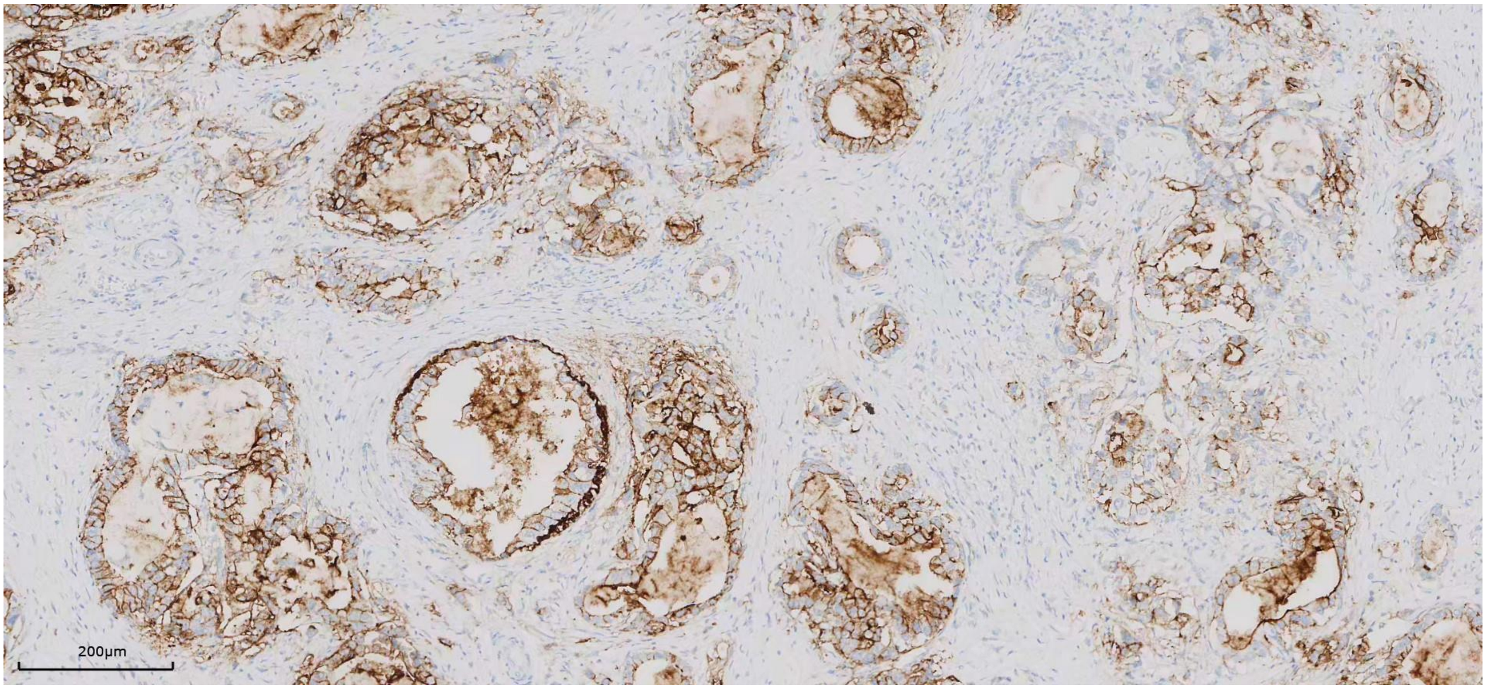

(B) Case meeting the ⩾75% threshold: ⩾2+ membranous CLDN18.2 staining in ⩾75% of tumor cells (original magnification, ×200).
